# Expression of p16 in Conjunctival Intraepithelial Neoplasia Does Not Correlate with HPV-Infection 

**DOI:** 10.2174/1874364100802010048

**Published:** 2008-03-28

**Authors:** Claudia Auw-Haedrich, Gottfried Martin, Helga Spelsberg, Rainer Sundmacher, Nikolaus Freudenberg, Philip Maier, Thomas Reinhard

**Affiliations:** 1 Eye Hospital, Albert-Ludwigs-University, Killianstr. 5, D-79106 Freiburg, Germany; 2 Eye Center, Clinic Pallas Otten, Switzerland; 3 Eye Hospital, Heinrich-Heine-University, Moorenstr. 5, D-40125 Düsseldorf, Germany; 4 Institute for Pathology, Albert-Ludwigs-University, Breisacherstr. 115a, D-79106 Freiburg, Germany

**Keywords:** p16, conjunctival intraepithelial neoplasia, HPV.

## Abstract

The aim of our study was to identify the frequency of expression of p16_INK4a_ (CDKN2A) and HPV (human papilloma virus) in different grades of conjunctival intraepithelial neoplasia (CIN).

Twelve specimens including CIN I (2), II (3), III (5), and CIN with beginning invasion (2), as well as 15 control specimens, were stained with antibodies against p16_INK4a_ and MIB1. The presence of HPV was examined by PCR.

p16 as well as MIB1 were significantly elevated in CIN compared to control specimens (p<0.01) without correlation with the differentiation grade. Only two cases with CIN grade 3 contained HPV 16.

As few control specimens also showed increased p16_INK4a_ expression, p16_INK4a_ seems not to be a very reliable marker for the exact determination of CIN. It could serve as an additional diagnostic tool besides the morphological characterization. Our study suggested that p16_INK4a_ elevation is not associated with HPV infection.

## INTRODUCTION

Conjunctival intraepithelial neoplasia (CIN) presents as a spectrum from simple dysplasia to carcinoma in situ. CIN commonly arises in the limbal region and particularly occurs in elderly males who have lived in areas exposed to high levels of UV-B radiation [1], an assumed main risk factor in addition to HPV16 [2], especially in persons with pale skin, pale iris and propensity to sunburn [3]. UV light is thought to cause DNA damage and the formation of pyrimidine dimers [4].

Recently our immunohistochemical analysis showed a significantly higher expression of p63 in CIN and carcinoma compared to the normal specimens without correlation with the differentiation grade and MIB-1-rate [5], indicating its independence from cell cycle progression. p63 is a homologue of the tumor suppressor gene p53 first cloned by Yang A *et al.* in 1998 [6], which functions instead as an oncogene [7]. Here we were interested in investigating another cell cycle marker (in addition to MIB-1) in CIN, which could be a promising candidate to increase diagnostic specificity and safety.

**Fig. (1).**
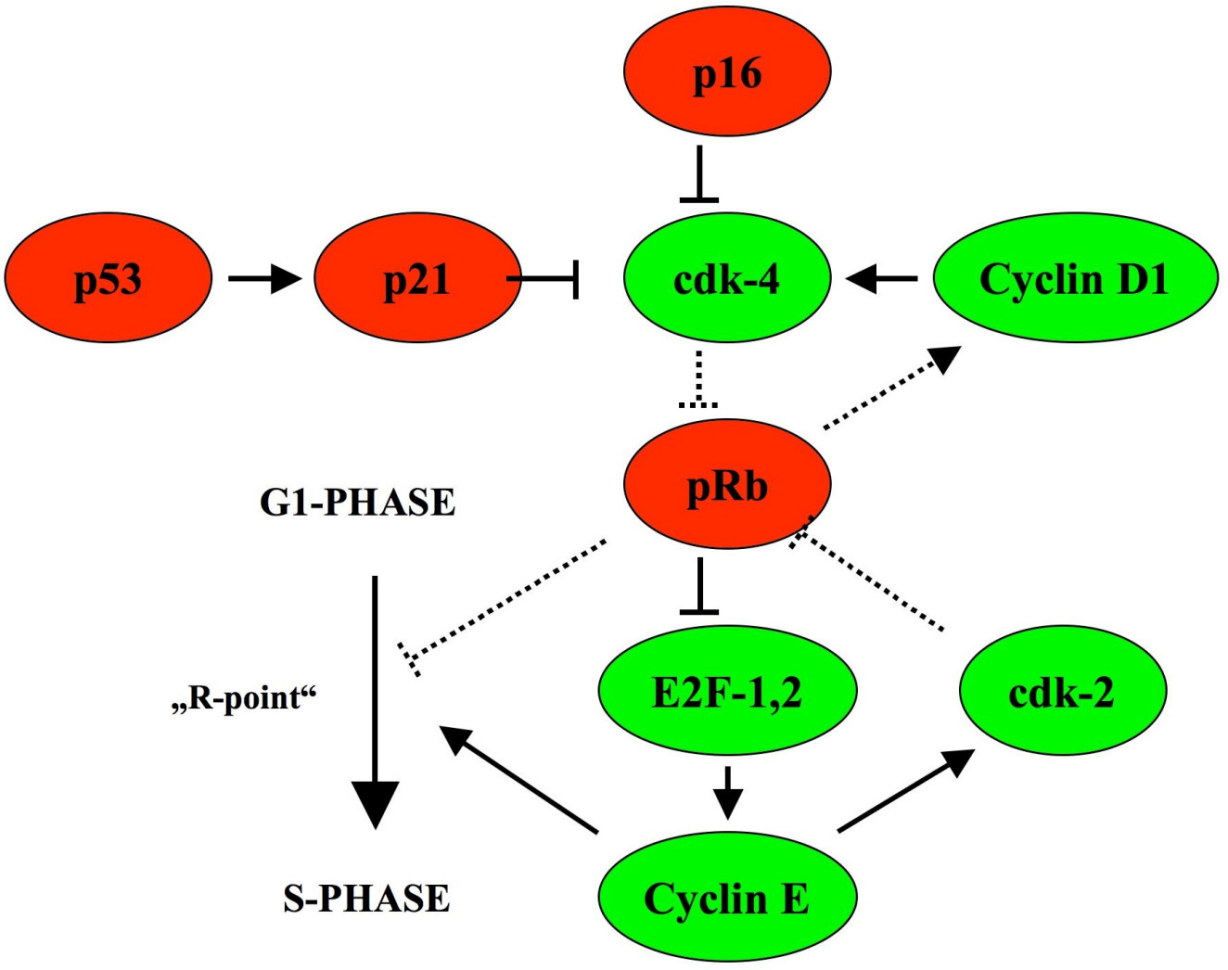
p16_INK4a_ restrains cell cycle progression by inhibiting the activity of CDK (cyclin dependent kinase) 4 and is upregulated in case cell cycle progression takes place due to phosphorylation of pRb (with permission of Graefes from [13]).

In cervical intraepithelial neoplasia, p16_INK4a_ (CDKN2A, cyclin-dependent kinase inhibitor 2A) was shown to indicate malignancy and progression into carcinoma [8, 9]. p16_INK4a_ restrains the progression of the cell cycle by inhibiting the activity of CDK (cyclin dependent kinase) 4, the latter targets pRb for phosphorylation and abolish pRb inhibition of E2F, driving cell cycle progression. In the presence of HPV infection, the HPV E7 protein binds to pRb and displaces E2F with subsequent E2F activation and cell cycle progression from the G1 to S phase and mitosis (Fig. **1**).

Due to pRb inactivation, p16 increases and becomes immunohistochemically detectable [10-12].

The aim of our study was to assess the frequency of expression of p16_INK4a_ and HPV in different grades of conjunctival intraepithelial neoplasia in order to find out if HPV-vaccination could also be important in order to prevent this disease.

## MATERIAL AND METHODS

The study was approved by the ethic comitee of the Albert-Ludwig University Freiburg, Germany, written informed consent was obtained from the study participants. 12 conjunctival specimens (see also Table **1**) excised from the bulbar conjunctiva with the suspicion of conjunctival intraepithelial neoplasia (CIN) and 14 macroscopically normal postmortem conjunctival specimens and 1 conjunctival specimen with slight inflammatory changes (postmortem time 18.2h in average, range 0-26.2h) were fixed in 4% formaldehyde in 0.075 M phosphate buffer for 24 h, dehydrated in increasing concentrations of ethanol (70%-99%) and infiltrated with paraffin (Merck, Darmstadt, Germany) at 60°C. Sections of 3 μm thickness were cut and floated on deionized water at 45°C, and single sections were mounted on Superfrost Plus glass slides (Menzel-Glaser, Germany). Slides were subsequently dried at 60°C for 1h. The haema-toxylin-eosin stained patient slides were diagnosed histologically as follows: 2 CIN grade I (up to 25% of the whole thickness of the specimen shows dysplasia), 3 CIN grade II (25-75% of the whole thickness of the specimen shows dysplasia), 5 CIN grade III (more than 75% of the whole thickness of the specimen shows dysplasia, this category also includes carcinoma in situ), and 2 CIN with beginning invasion (showing minute interruption of the basal lamina and dysplastic cells below the basal lamina level). The 15 macroscopically normal conjunctival specimens and 12 CIN specimens were stained immunohistochemically using the avidin biotin method including antigen retrieval using a microwave oven for 10 minutes at pH 6.0, before the mouse monoclonal antibodies p16^INK4a^ Ab-4 (clone 16P04 /JC2, Lab Vision Corporation, Fremont, USA) and Ki-67 (clone MIB-1, DAKO, Glostrup, Denmark) were applied for 2h. The negative controls used only showed occasional and slight cytoplasmic background staining. Cells that were considered positive for either p16^INK4a^ or MIB-1 clearly showed a reddish stained nucleus, and some displayed additional cytoplasmic positive staining.

At least 500 cells per specimen of representative vertical sections of the specimens at a magnification of 200x were counted, and the proportion of positively stained cells for each antibody was calculated. Statistical calculation was performed using the Kruskal-Wallis non-parametric test.

### HPV Detection by PCR

DNA from 1-3 5 µm paraffin sections was extracted as follows: paraffin was removed by xylol and the sections were recovered by centrifugation and drying. 100 µl of 100 mN NaOH were added and the sections were incubated for 1 h at 95°C. After the samples cooled, the solution was neutralized by adding 11 µl of 1 M NaH2PO4. 1-2 µl were used in a 20 µl PCR reaction containing 200 µM dNTPs, 500 nM primer mix PGMY11 (GCACAGGGACATAACAATGG, GCGCAGGGCCACAATAATGG, GCACAGGGACATAA TAATGG, GCCCAGGGCCACAACAATGG, GCTCAGG GTTTAAACAATGG [14], 500 nM primer GP6+ (GAAAA ATAAACTGTAAATCATATTC [15], and 2 U Taq. Our primers, taken from the literature, bind at the conserved region of the L1 gene. The PCR program was 2 min incubation at 94°C, 40 cycles of 94°C for 30 s, 55°C for 30 s, and 72°C for 30 s, and 5 min incubation at 72°C. ß-actin (CTACAATGAGCTGCGTGTGG, CGGTGAGGAT CTTCATG AGG) was used as a control. 2 µl of the PCR product were diluted to 100 µl, of which 2.5 µl were used for the second PCR with the primers GP5+ (TTTGTTACTGT GGTAGATACTAC) and GP6+. The second PCR reaction was adjusted to an annealing temperature of 40°C for 60 s and elongation at 72°C for 90 s. Fragments of approximately 132 bp were isolated from the agarose gel and cloned into pBluescript (Stratagene, Amsterdam, The Netherlands). At least 3 clones were selected for each fragment and sequenced. All sequences showed a HPV fragment flanked by vector sequences. Sensitivity of the PCR was checked with a sample known to contain HPV. As we used nested PCR, we had a very sensitive as well as specific detection system which was commonly reported in the literature. In situ hybridization would have been an important supplementary method to detect HPV, which could not be performed due to lack of tissue in most of our CIN cases.

## RESULTS (TABLES 2 AND 3)

### p16 Expression in Normal Conjunctival Specimens

**Fig. (2).**
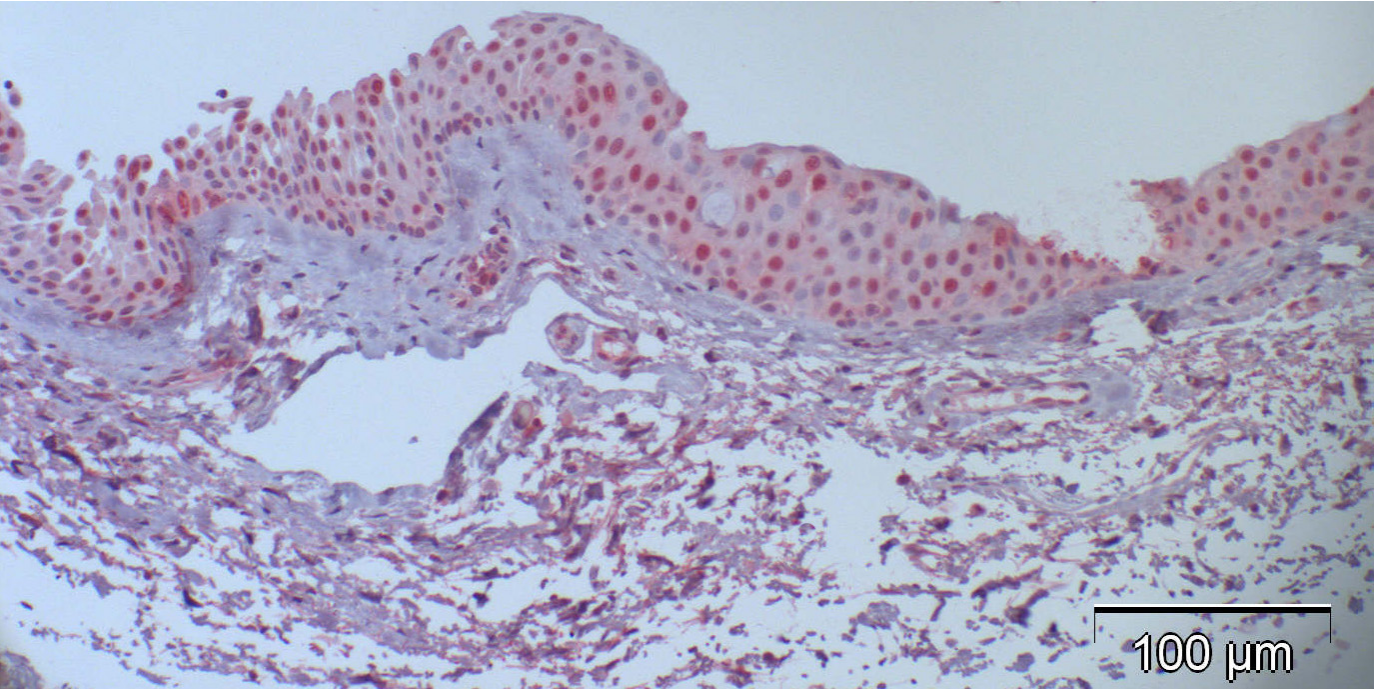
Case 5 from Table **2** (normal control): 27% are p16-positive cells, located throughout all epithelial layers with stronger staining of the basal and parabasal layer compared to the more superficial cell layers; some stromal cells show heavy background staining.

**Fig. (3).**
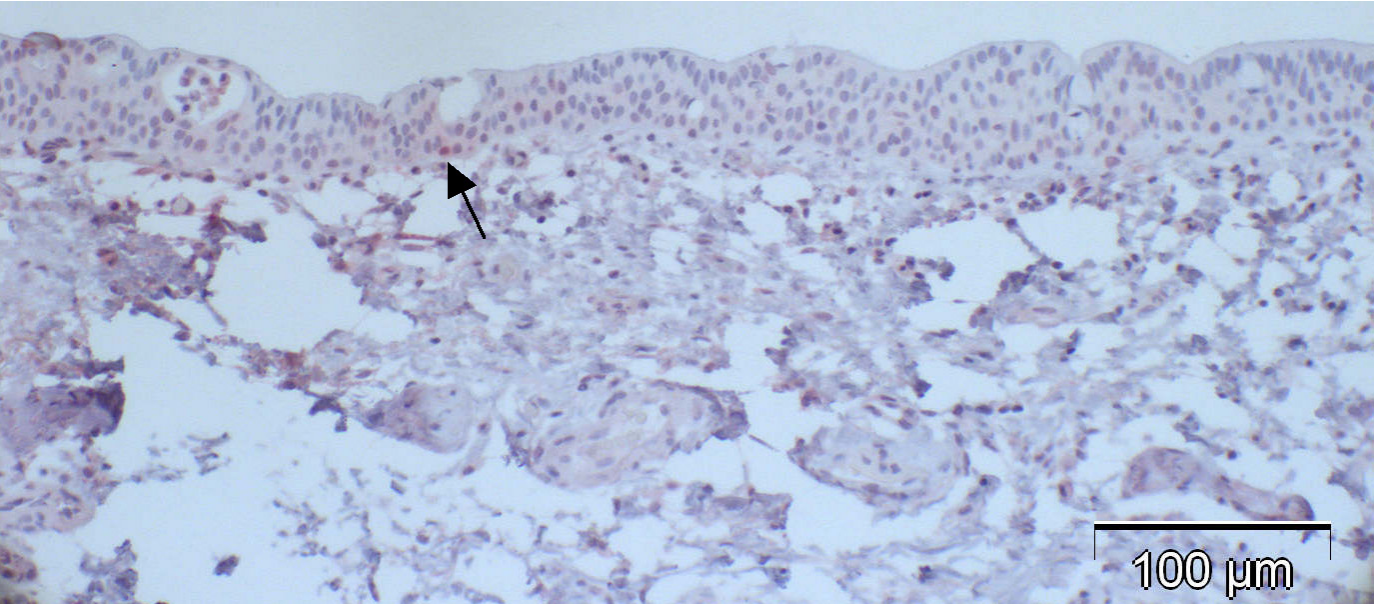
Case 2 from Table **2** (normal control): almost negative p16 expression despite positive staining for p63 in a previous study [5] indicating functioning immunoreactivity of the specimen (arrow: positive nucleus).

Most of the normal conjunctival specimens showed stronger p16 expression in the basal layers, compared to the more superficial cell layers (Fig. **2**); few specimens were almost or completely negative for p16 expression (Fig. **3**). The proportion of p16-positive cells in the normal postmortem conjunctiva and the conjunctival specimen with inflammatory changes varied between 0 and 51.8%, and the mean was calculated at 16.4%.

### p16 Expression in CIN

In contrast to observations in normal specimens, many of the CIN specimens stained positive for p16, exhibiting a similar trend to that in normal specimens, with a stronger intensity in the basal layers compared to the superficial layers (Fig. **4a-d**).

The percentage of p16-positive cells in the CIN specimens was higher than that in normal specimens, and varied between 27.2 and 83.9%, with the mean at 53.6%. Notably, p16 expression was significantly elevated in CIN samples compared to the control specimens (p<0.001, Fig. (**5**)). No correlation was found between p16 expression and the differentiation grade (correlation coefficient 0.2).

### MIB-1 Expression in Normal Conjunctival Specimens

Analysis of MIB-1 expression revealed that the control specimens only contained 0 and 0.2% MIB-1 positive cells among all specimens. Neither the expression of p16 nor MIB-1 seemed to depend on the postmortem time, which ranged between 0 and 26.2 hours (mean of 18.2 hours).

### MIB-1 Expression in CIN

**Fig. (6).**
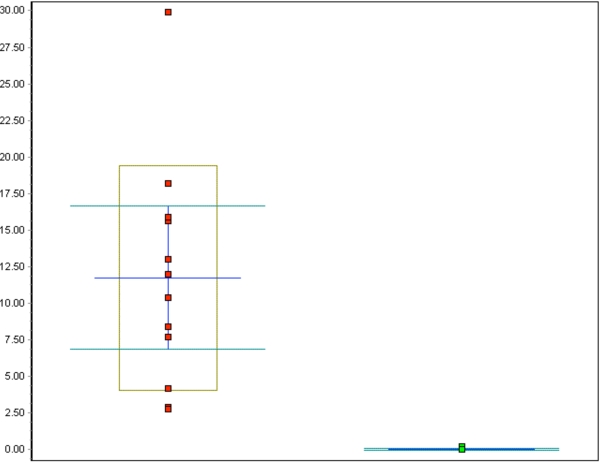
Whisker plot of all Ki-67 counts in %: red dots represent the CIN cases, green dots represent the control cases, p<0.001.

Analysis of CIN samples revealed that MIB-1 expression was elevated in the dysplastic specimens, though not to the extent of p16 expression (Fig. **6**).

The percentage of MIB-1 positive cells varied between 2.8 and 29.9%, the mean being 11.8%, a significant increase compared to the control specimens (p<0.001). Neither a correlation between differentiation grade and MIB-1 positive staining (correlation coefficient 0.2) nor between p16 and MIB-1 expression (correlation coefficient 0.4) was found.

### HPV Positivity in Normal Conjunctival Specimens and CIN

None of the 15 control specimens were found to contain HPV, while β-actin expression was detected in 14 of the controls. Only 2 of the CIN specimens (case 6 and 8) contained HPV type 16, and both were classified as grade III; eleven of the CIN specimens were found to express β-actin.

## DISCUSSION

Cervical intraepithelial neoplasia has been shown to be associated with HPV-infection in more than 99% of the cases [16, 17]. Infection of “high risk” strains, such as HPV 16, 18, 31, 33, 52, and 58, is associated with more intense p16 expression in the infected cervical epithelial cells compared to “low risk” strains like HPV 6 or 11 [18]. Recent biological studies have revealed that p16 expression is markedly influenced by the status of Rb expression, and p16 overexpression has been demonstrated in cervical cancers, presumably due to the functional inactivation of Rb by human papillomavirus (HPV) E7 protein [18].

Our study shows increased p16 expression in conjunctival intraepithelial neoplasia compared to normal conjunctival specimens. This result is in contrast to that of Kuo *et al.* [19], whose CIN specimens were negative for p16 expression. Jung *et al.* found p16 elevated in conjunctival squamous cell carcinoma [20], which can develop from conjunctival intraepithelial neoplasia studied here. We assume that postmortem alterations of the control specimens with subsequent decreased immunoreactivity are not the reason for the significant elevation of p16 in CIN compared to the controls. Few control specimens showed even surprisingly high p16 expression despite their average or even higher than average postmortem time. Nevertheless, no correlation between p16 expression and differentiation grade was found, in contrast to the results described by Klaes *et al*. in cervical intraepithelial neoplasia [12]. This is possibly due to the low number of cases included in our study. The fact that few control specimens showed high p16 expression supported the hypothesis that this could be due to HPV-infection with pRb inactivation (see Fig. (**1**)) and subsequent p16 elevation. However, our results indicating the control specimens as HPV negative did not support the above-mentioned hypothesis. Furthermore, only 2 of the 12 CIN specimens turned out to be HPV positive, with type 16 detected. This is in line with the results of Tulvatana *et al*. who examined 30 CIN specimens and found negative HPV and positive β-globin DNA in 16 cases [21], and Eng *et al*., who did not find any HPV in 20 malignant epithelial conjunctival tumours [22]. In contrast, Scott *et al*. found HPV in 10 CIN cases (5 with HPV 16 and 5 with HPV 18) with negative results in the control specimens. In each of those CIN specimens, 20%–40% of dysplastic cells expressed HPV E6 [23]. These studies show that the etiology of CIN is not homogenous and in addition to HPV, other pathogens such as UV-radiation may play a role in CIN development.

In most of the cases presented here, p16 elevation was not found to be HPV-induced, but seems to be caused by another agent with or without the involvement of pRb status change. p16 did not correlate positively with differentiation grade and MIB-1-positivity, which might indicate that in some cells p16 elevation successfully prevented cell cycle progression beyond the restriction point.

In conclusion, p16 seems to be a malignancy indicator in conjunctival epithelial tumours, especially towards CIN, but caution must be applied as normal conjunctiva also occasionally displays high p16 expression. Our study shows that HPV is not the only oncogenic agent in CIN etiology and HPV vaccination seems not to prevent CIN in future.

## Figures and Tables

**Fig. (4a).**
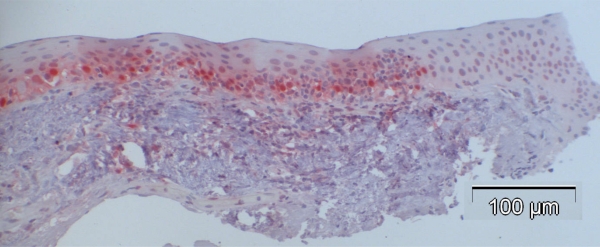
Case 2: CIN Grade I with dysplasia including the lower 25% of the whole epithelial thickness with p16-positivity of 51%.

**Fig. (4b).**
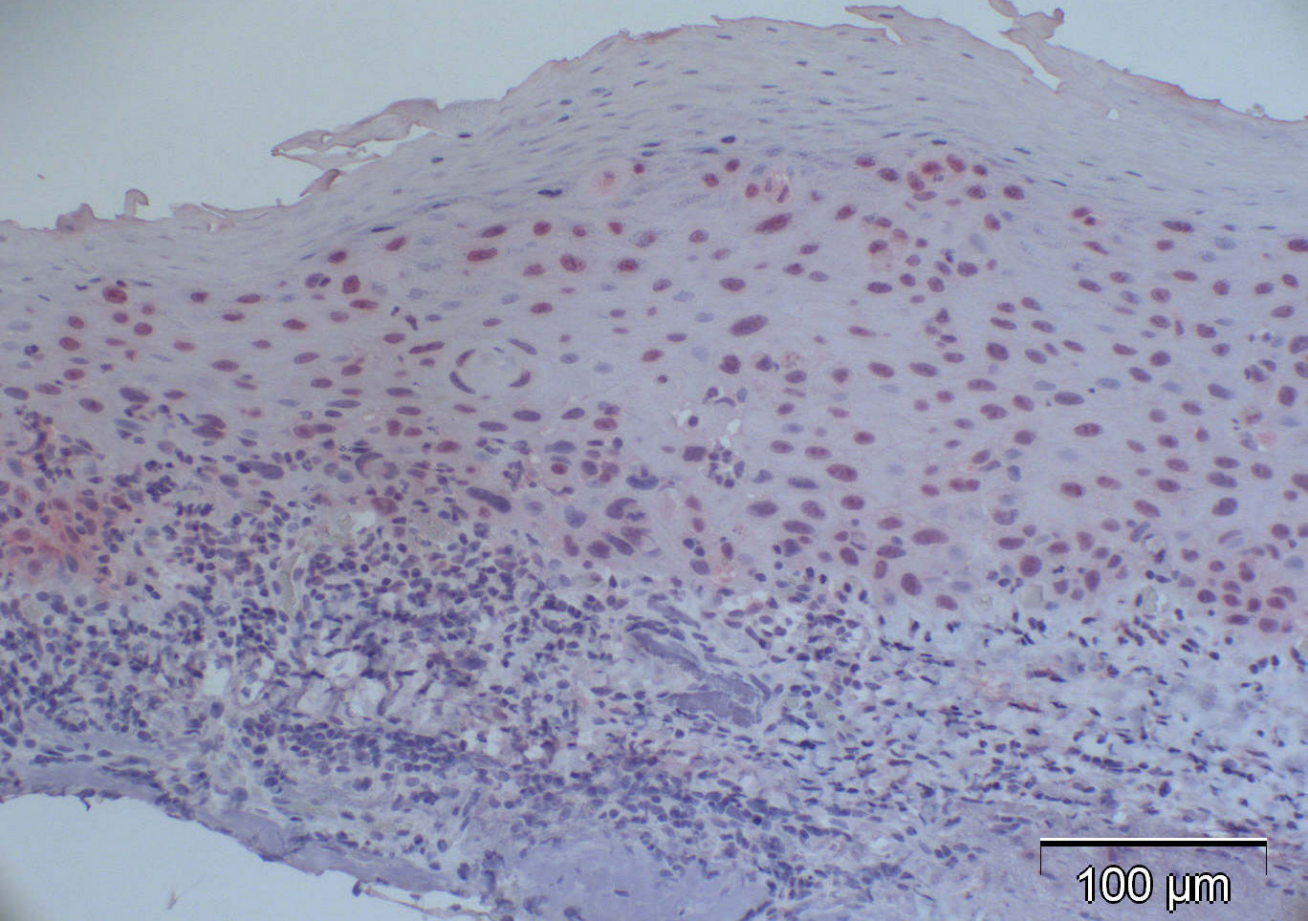
Case 3: CIN Grade II with dysplasia including the lower 75% of the whole epithelial thickness with p16-positivity of 57%.

**Fig. (4c).**
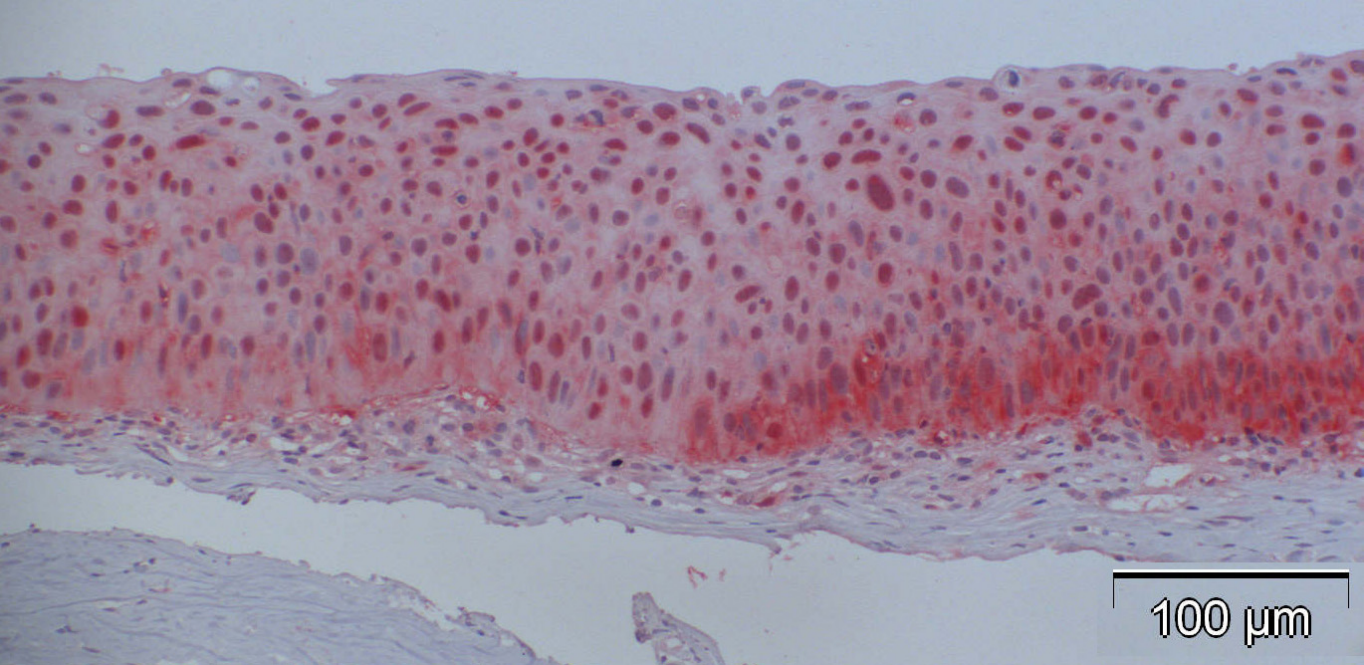
Case 8 from Table **3** (CIN III): 84% of the cells showed positive nuclear staining, and many of them also displayed cytoplasmic staining for p16 with higher intensity of basal compared to the more superficial layers.

**Fig. (4d).**
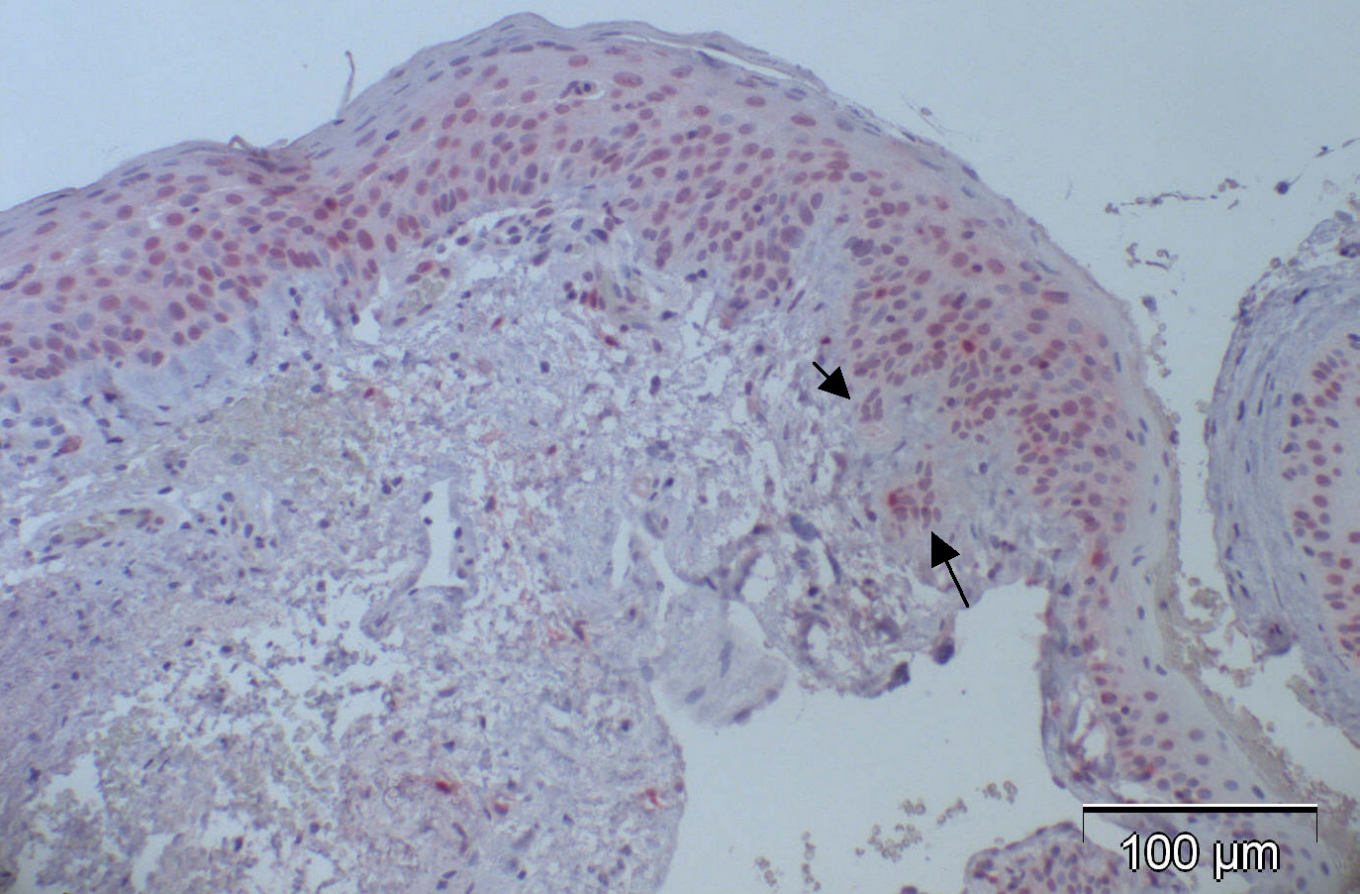
Case 12 from Table **3** (CIN with beginning invasion, arrows): 37% of the cells showed positive nuclear staining.

**Fig. (5).**
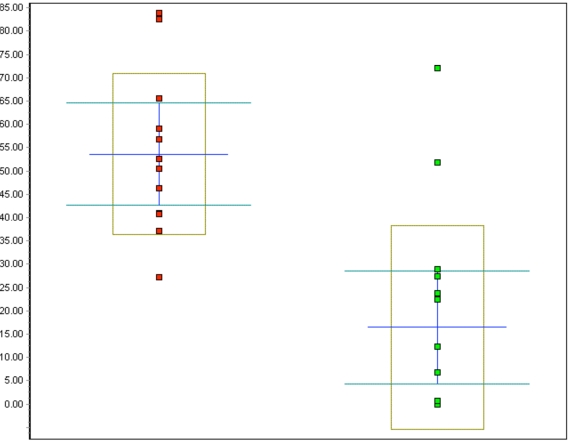
Whisker plot of all p16 counts in %: red dots represent the CIN cases, green dots represent the control cases, p<0.001.

**Table 1.** Clinical Data of the CIN Specimens

No	Age	Gender	Clinical Findings	Treatment Prior to Excision	Risk Factors	Proposed Pathogenic Sequence	Tumor Location
1	79	f	Chronic conjunctivitis	Cromoglycic acid	None	Primary CIN?	Limbus, 16x6x4mm
2	67	m	Conjunctivitis	None	None	Primary CIN?	Nasal and temporal limbus
3	39	m	Red eye	Vit A ointment	None	Primary CIN?	Temporal limbus
4	73	m	Corneal neoplasia	None	None	Possibly primary CIN developing from a pinguecula	Corneal at the limbus, adjacent to pinguecula
5	61	m	Corneal neoplasia	None	None	Primary CIN?	Medial limbus with corneal part (2-3mm) 10x5x2mm
6	64	m	At least 4 years ocular surface problems with circular pannus	Topical steroids, antiviral ointment	HPV 16	Malignant transformation due to HPV 16	Limbus with corneal part at 7 and 12h
7	67	m	"Pterygium" since at least 3 years with unusual extension	None	None	Primary CIN mimicking pterygium or CIN developing from altered epithelium of a pterygium	Medial limbus with corneal part (2mm)
8	56	f	Atypical Pterygium with whitish plaque and vascularisation	None	HPV 16	Malignant transformation due to HPV 16	Limbus, 11x7x1mm
9	61	m	Red eye	None	None	Primary CIN?	Medial limbus with corneal part (2mm)
10	53	m	Superficial vascularisation onto the cornea	None	Explosion injury 30 years previously	DNA-damage due to the trauma and malignant transformation during repair phase	Limbus with corneal part (2mm)
11	29	m	Whitish neoplasia	1991 tectonic keratoplasty due to malignancy, 1992 rekeratoplasty due to corneal melting	Xeroderma pigmentosum, UV-B radiation	DNA-damage and malignant transformation due to UV-B radiation and defective repair mechanisms	Limbus with corneal part (4mm) 4x4x2
12	67	m	Red eye	Dexpanthenol eye drops	None	Primary CIN?	Medial limbus

**Table 2.** Normal Bulbar Conjunctival Specimens and the Proportion of p16 and MIB-1 Positive Cells

No	p16-Positive Cells	MIB-1-Positive Cells	HPV	β-Actin	Postmortem Time h=hours, ‘=min
1	6.8% (37/545)	0% (1/500)	-	+	23h 40'
2	0.1% (1/751)	0% (0/500)	-	+	<24h
3	29.0% (228/785)	0% (0/500)	-	+	26h 10'
4	0.0% (0/750)	0% (0/500)	-	+	23h 21'
5	27.3% (241/883)	0% (0/500)	-	+	20h 30'
6	0.0% (0/750)	0% (0/500)	-	+	22h
7	0.0% (0/750)	0% (0/500)	-	+	21h 3'
8	51.8% (309/597)	0% (1/500)	-	+	21h 48'
9	0.0% (0/500)	0% (0/500)	-	-	<24h
10	0,0 (0/500)	0% (0/500	-	+	10h
11	22.5% (120/534)	0% (1/500)	-	+	15h 5’
12	72.1% (382/530)	0% (0/500)	-	+	20h 20’
13	12.4% (71/571)	n.a.	-	+	0h (biopsy)
14	0.6% (3/503)	n.a.	-	+	15h 30’
15	23.8% (121/509)	n.a.	-	+	24 h
Mean ± SD	16.43 ± 21.87	0.033 ± 0.078			18.2 ± 7.1

**Table 3.** Differentiation Grade of the CIN Specimens and the Proportion of p16 and MIB-1 Positive Cells (Grade 4 = CIN with Beginning Invasion)

No.	Grade	p16-Positive Cells	MIB-1-Positive Cells	HPV	β-Actin
1	1	66% (402/614)	3% (15/510)	-	+
2	1	51% (263/521)	4% (22/522)	-	+
3	2	57% (312/550)	13% (72/552)	-	+
4	2	46% (258/558)	16% (89/569)	-	+
5	2	41% (320/782)	10% (72/694)	-	+
6	3	53% (378/720)	16% (120/754)	+ (type 16)	+
7	3	41% (251/615)	8% (70/831)	-	+
8	3	84% (536/639)	18% (118/649)	+ (type 16)	+
9	3	59% (395/669)	30% (183/613)	-	+
10	3	83% (598/725)	12% (60/502)	-	+
11	4	27% (149/547)	3% (15/539)	-	n.a.
12	4	37% (247/664)	8% (42/546)	-	+
Mean ± SD		53.6 ± 17.3	12% ± 8%		
